# Superhydrophobic and oleophobic microtextured aluminum surface with long durability under corrosive environment

**DOI:** 10.1038/s41598-023-28587-z

**Published:** 2023-01-31

**Authors:** Olatz Adarraga, Cecilia Agustín-Sáenz, Izaskun Bustero, Fabiola Brusciotti

**Affiliations:** TECNALIA, Basque Research and Technology Alliance (BRTA), Parque Científico Y Tecnológico de Gipuzkoa, Mikeletegi Pasealekua, 2, 20009 Donostia-San Sebastián, Spain

**Keywords:** Chemistry, Engineering, Materials science

## Abstract

Superhydrophobic (SHP) and oleophobic aluminum surfaces have been prepared through the combination of a scalable chemical microtexturing process and surface functionalization with long-chained polyfluoroalkyl moieties. The effect of an anodic layer on the microtextured surface has been assessed considering surface morphology, superhydrophobicity, surface mechanical properties and corrosion protection enhancement. The surface functionalization with polyfluoroalkyl moieties has been tackled in two different ways: (i) grafting of the polyfluoroalkyl moieties and (ii) deposition of a thin hybrid coating with low content of polyfluoroalkyl-containing compound. Aluminum surfaces showing high durability in salt spray environments, which maintain SHP and oleophobic properties at least up to 2016 h have been attained. Applications for this kind of surfaces range from easy-to-clean surfaces to anti-icing or anti-condensation functionalities that could be of interest for several sectors.

## Introduction

Aluminum alloys are widely used in a large variety of sectors including construction, automotive, marine, aeronautics or home appliances thanks to their excellent properties, such as superior high-specific strength, remarkable electrical conductivity or relatively low-specific weight. In recent years, the request of surfaces with new properties such as self-cleaning^1^ or anti-icing^2,3^ also exhibiting high corrosion resistance^4^ has guided research into new surface treatments showing superhydrophobicity, i.e., water contact angle (WCA) higher than 150° with sliding angles lower than 5°^5^ and oleophobicity, i.e., contact angle higher than 90° with low energy liquids like hexadecane.

Superhydrophobicity of a surface is ruled by both its composition and morphology. The maximum WCA of low interfacial energy smooth surfaces can hardly achieve 110–120°. Therefore, in order to achieve superhydrophobicity, the surface must combine chemical and morphological characteristics, as studied by many authors^5,6^. Thanks to the tailoring of specific surface roughness (micro-nanotexturing), the hydrophobicity of a surface with low interfacial energy can be further increased (Cassie-Baxter to Wenzel state), leading to superhydrophobicity. Different methods are reported in the literature to tailor the surface roughness such as mechanical processing^7^, chemical etching^8–10^, electrochemical processing^11^, laser texturing^12^ or anodizing^13^. However, the implementation of some of them at an industrial level is challenging due to relatively high processing times and costs or difficulty to treat non-flat pieces or complex geometries. Furthermore, the applied method can affect material properties such as mechanical, durability or corrosion resistance.

Considering that cost-effectiveness and homogeneous properties in complex geometry pieces are required by industry, several chemical processes allowing large scale manufacturing are considered potentially suitable for superhydrophobic (SHP) and oleophobic aluminum parts production.

Among the publications addressing chemically processed aluminum SHP surfaces, only few of them study the oleophobicity^10^. Surfaces repelling both water (hydrophobic) and oil (oleophobic), called amphiphobic, are more difficult to process than surfaces with merely hydrophobic properties^14^. For instance, Choi et al.^15^ obtained SHP and oleophobic hierarchical aluminum surfaces with different morphologies using three different kinds of alkaline-based chemical etching processes. Carneiro et al.^16^ achieved SHP and oleophobic aluminum surfaces through chemical etching followed by the deposition of organically modified silicate coatings synthesized by sol‐gel methods. Varshney et al.^17^ achieved SHP aluminum surfaces with self-cleaning and antifog properties through chemical etching and passivation with Lauric acid. Ruan et al.^18^ obtained different SHP aluminum surfaces with anti-icing functionality through a specific electrochemical anodic oxidation and chemical etching methods that simplify the fabrication procedures for the attainment of SHP surfaces. Barthwal et al.^19^ fabricated a mechanically stable superamphiphobic aluminum, i.e., WCA and hexadecane contact angle (HCA) higher than 150°, by combining simple chemical etching and anodization with 1H,1H,2H,2H-perfluorooctyltrichlorosilane (FAS13) grafting. More recently, Kikuchi et al.^20^ produced a superamphiphobic (water and dodecane contact angles were higher than 150°) aluminum surface combining electrochemical etching (in hydrochloric acid solution) and anodizing (in pyrophosphoric acid solution) methods demonstrating high contact angles with different sliding angles depending on the anodizing time.

However, to make a qualitative jump into industrial applications, durability of SHP and oleophobic performance on microtextured aluminum surfaces is one of the major concerns addressed in recent works^14^. SHP and oleophobic surface processing and ageing is explored in literature with the objective to increase durability as well as avoiding corrosion, especially in moist environments. Zheng et al.^21^ fabricated a SHP aluminum surface through anodization in sulfuric acid electrolyte, followed by surface modification with myristic acid, showing durable SHP performance after sandblasting (during 60 s) and several UV/water condensation cycles. Zhao et al.^22^ reported a SHP aluminum alloy surface (AA5052) obtained by electrodeposition of Ni–Co coating whose interfacial energy was reduced after treatment with 6-(N-allyl-1,1,2,2-tetrahydroperfluorodecyl)amino-1,3,5-triazine-2,4-dithiol monosodium which exhibited durable WCA without corrosion signs after 4 weeks in air exposure. Yin et al.^23^ generated SHP aluminum surface by anodization process and chemical modification by myristic acid. The results revealed that the corrosion was effectively inhibited by the formation of a stable SHP film with little variation in WCA after 24 h of immersion in seawater. Wang et al.^24^ obtained SHP aluminum surfaces by combination of chemical etching (in hydrochloric acid solution), high field anodizing and grafting with 1H,1H,2H,2H-perfluorooctadecyltrichlorosilane (FAS33), that showed long term stability of the SHP performance after 24 h immersed under water and 72 h impacted by freely falling water droplets. They demonstrated its good corrosion resistance through electrochemical testing.

Barthwal and Lim^25^ produced a SHP aluminum alloy surface (AA6061) with a double scale roughness through chemical etching which was covered with polydimethylsiloxane by vapor deposition keeping superhydrophobicity after immersion for 7 days in a highly saline solution. The durability of the surface was also studied by exposing the fabricated sample in air for 8 months, showing excellent long-term stability at these conditions. Li et al.^26^ produced SHP aluminum surface by chemical etching and cyclic assembly using hydrochloric acid, phytic acid, cerium (III) chloride or ferric chloride and 1H,1H,2H,2H-perfluorooctyltriethoxysilane (FAS13). Such SHP surfaces demonstrated thermal stability up to 200 °C and chemical stability up to 16 h, immersed in 3.5% sodium chloride solution.

For a wide range of targeted applications of modified aluminum surfaces like self-cleaning, anti-icing, antifouling, and others, studies addressing representative long-term durability or ageing of wettability properties of tested surfaces are needed before these solutions can go into market. In this work, a chemically microtextured aluminum surface (AA1050) has been obtained by chemical etching in hydrochloric acid solution. To enhance corrosion resistance and mechanical properties of etched surfaces, sulfuric anodic layers have been grown onto the chemically microtextured surfaces. Then a silane with a non-hydrolysable long polyfluoroalkyl group, 1H,1H,2H,2H-perfluorodecyltriethoxysilane (FAS17), has been used to modify the obtained AA1050 surface in two ways: (i) self-assembled monolayer (SAM) by FAS17 grafting and (ii) deposition of a thin hybrid (methacrylate, silica and zirconia based) sol–gel coating containing FAS17 in its formulation. Compared with previous works, in this one we have obtained a scalable, simple and cost-effective chemical microtexturing process combined with industrial anodic layers and polyfluoroalkyl functionalization leading to SHP and oleophobic surfaces, demonstrating high durability in corrosive environments.

## Results and discussion

### Surface morphology and roughness

**Figure 1:**
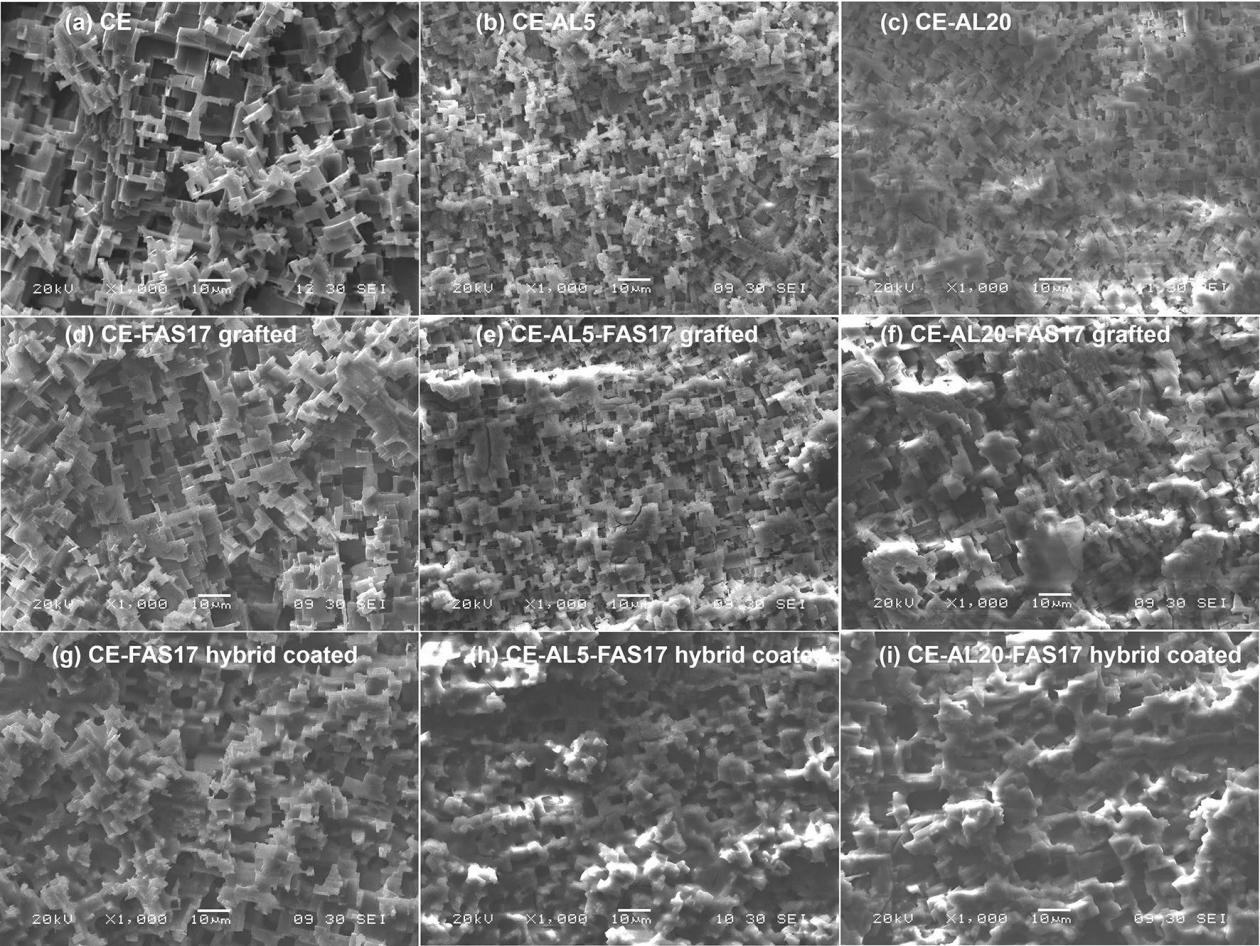
SEM images of (**a**) CE surface, (**b**) chemically etched and anodized surfaces CE-AL5, (**c**) chemically etched and anodized CE-AL20, and functionalized samples (**d**) CE-FAS17 grafted, (**e**) CE-AL5-FAS17 grafted, (**f**) CE-AL20-FAS17 grafted, (**g**) CE-FAS17 hybrid coated, (**h**) CE-AL5-FAS17 hybrid coated and (**i**) CE-AL20-FAS17 hybrid coated.

**Figure 2:**
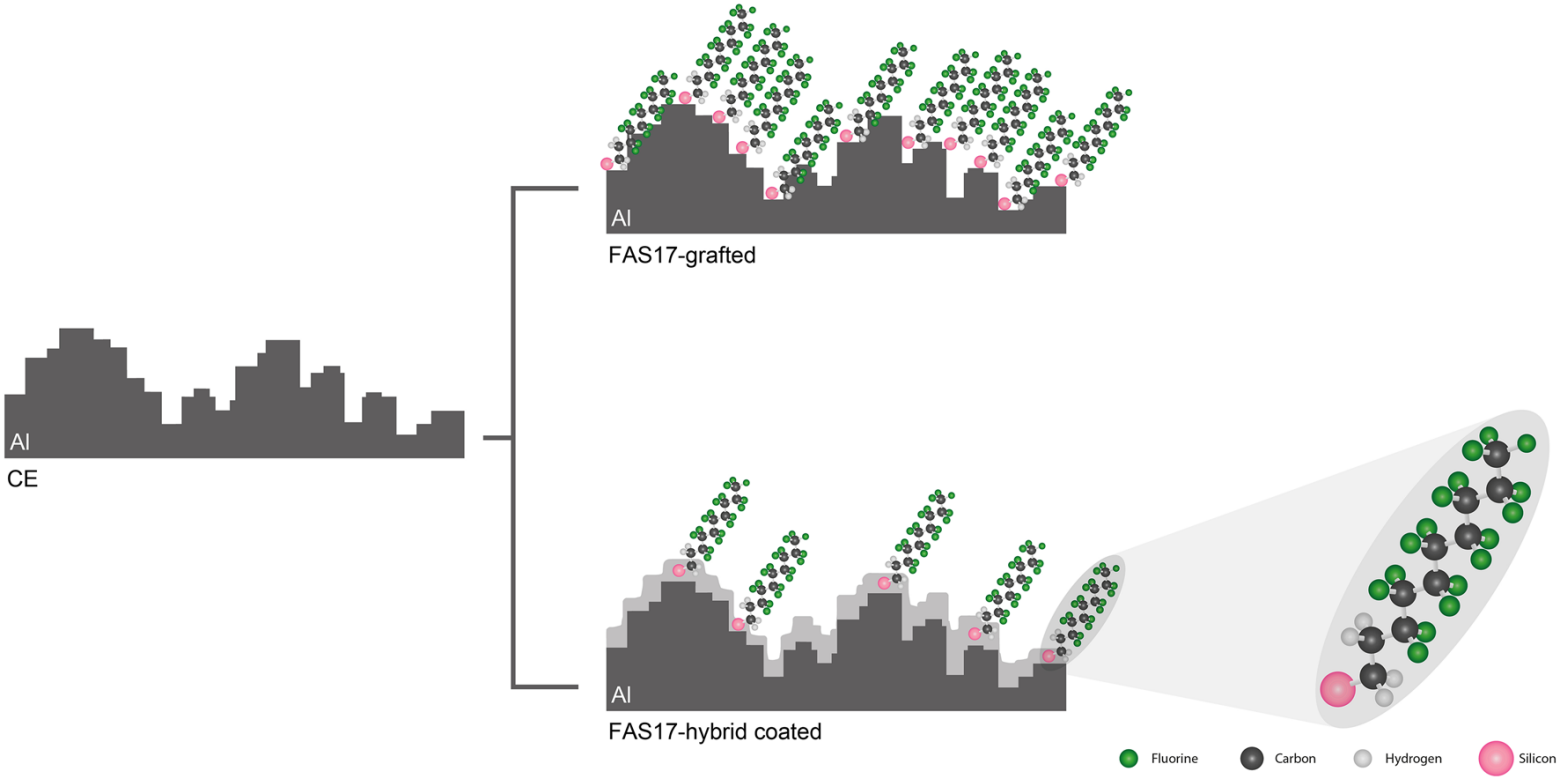
Schematic diagram of micro-step like structure and FAS17 modifications.

The chemical etching process produced a micro-step like texture resulting from the reaction between HCl and the surface of crystalline aluminum. The appearance of the surface of the AA1050 after chemical etching is presented in Fig. 1a. The large amount of dislocation defects in the crystalline surface acts as preferential sites for reacting with some specific dislocation etchants such as HCl^10^. This reaction begins producing pits that spill over the surface, and by progressively interconnecting them, a homogeneous micro-step like roughness is obtained all over the aluminum surface^19^. This micro-step like texture is schematically represented in Fig. 2. A minimum reaction duration is required to achieve homogeneous micro-step structure. Through visual inspection, the optimum reaction duration for a complete surface etching was fixed between 17 and 24 min. In this interval, apparently, the surface was completely and homogenously etched, and a homogeneous mate finish was observed.

**Figure 3:**
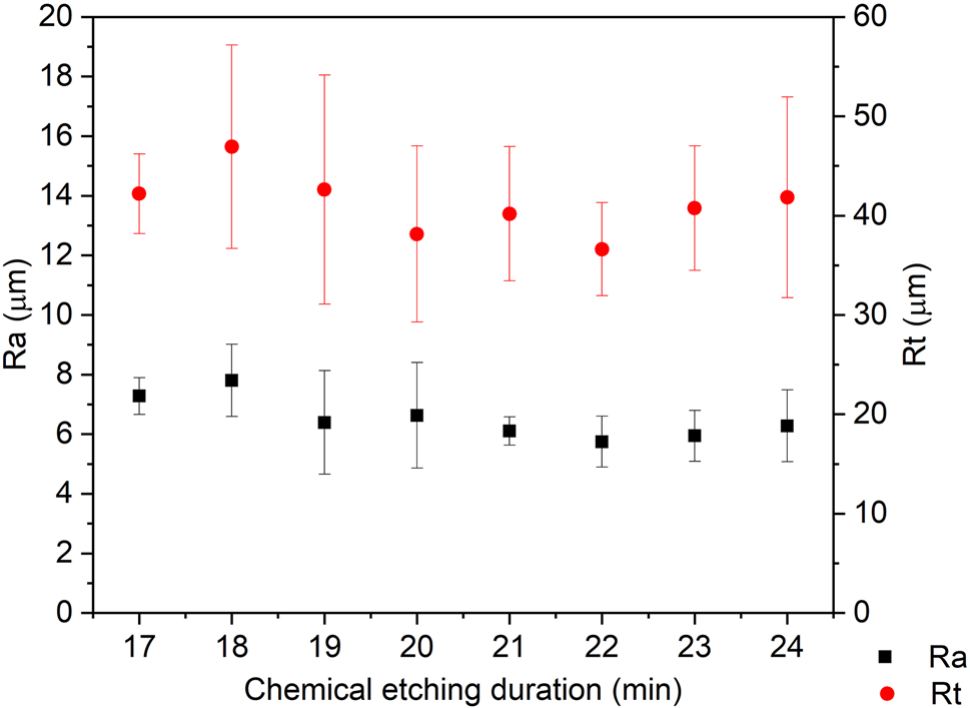
Roughness (Ra and Rt) measurement on chemically etched aluminum samples with different reaction duration.

**Figure 4:**
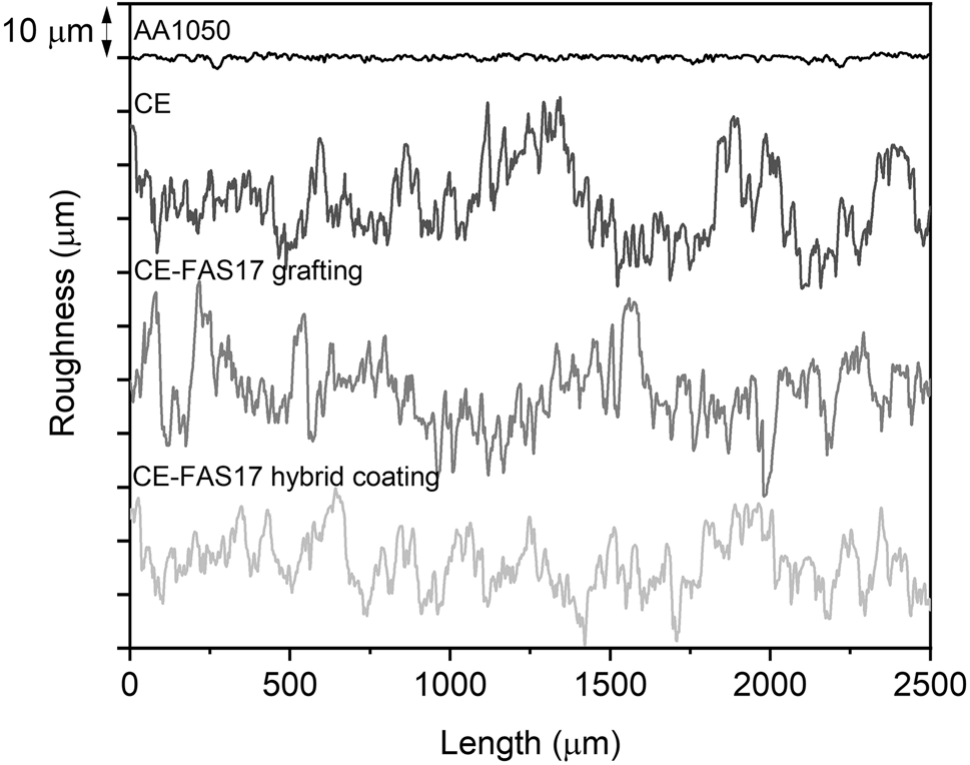
Surface profile of pristine AA1050, chemical etched AA1050 surface (CE), FAS17 grafted (CE-FAS17 grating) and FAS17 hybrid coated (CE-FAS17 hybrid coating) surfaces after 22 min of chemical etching reaction.

The arithmetic average roughness (Ra) of pristine AA1050 was 0.36 ± 0.02 µm and the maximum height of the profile (Rt) was 2.84 ± 0.18 µm (see Table 1). After chemical etching in 3.0 M HCl between 17 and 24 min, mean Ra of the surfaces was between 5 and 7 µm and mean Rt was between 35 and 47 µm, as presented in Fig. 3. From these measurements, it was observed that Ra of the surfaces etched at shorter duration showed higher Ra values, ⁓ 7 µm, and large dispersion, which was attributed to an incomplete surface processing, presenting both pits and un-etched areas. Etching duration longer than 19 min resulted in surfaces with lower Ra values, ⁓ 6 µm, and from 21 min onwards Ra dispersion was lower, which was related to the homogenous surface obtained. Concerning Rt similar average results were observed for the different samples, close to 40 µm, although slightly larger dispersion was observed at shorter etching duration. From 21 min duration onwards Rt dispersion was lower. For the 24 min reaction, Rt dispersion values increased probably due to an initial over-etching process of the alloy. The Fig. 4 shows the profile of AA1050 before and after 22 min of reaction (CE) and Ra and Rt are presented in Table 1.

**Table 1:** Roughness results on pristine AA1050, as-prepared chemical etched and anodized AA1050; after FAS17-grafted and FAS17-hybrid coating treatments.

	As-prepared		FAS17-grafted		FAS17-hybrid coated	
	Ra (µm)	Rt (µm)	Ra (µm)	Rt (µm)	Ra (µm)	Rt (µm)
AA1050	0.36 ± 0.02	2.84 ± 0.18	–	–	–	–
CE	5.75 ± 0.85	36.62 ± 4.69	5.99 ± 1.39	38.40 ± 2.44	4.32 ± 0.68	31.23 ± 2.36
CE-AL5	4.98 ± 0.39	36.86 ± 4.30	5.09 ± 0.48	35.27 ± 4.03	3.40 ± 0.20	24.90 ± 1.65
CE-AL20	5.69 ± 0.71	39.91 ± 6.59	4.98 ± 0.67	36.02 ± 7.52	4.31 ± 0.71	31.55 ± 6.96

The surfaces etched during 22 min were subjected to two types of post-processes, (i) anodization and (ii) introduction of polyfluoroalkyl moieties, with the aim to improve durability and amphiphobicity.

Anodizing process produces a thick aluminum oxide layer on the surface of raw aluminum through an electrochemical anodic reaction. The structure of the anodized aluminum oxide layer is nanoporous, formed by a hexagonal array of cells with cylindrical pores with a diameter of 25 nm to 0.3 μm and depth up to 100 μm^27^. The combination of chemical etching and anodization processes is expected to produce the combination of micro and nanostructures and the attainment of a hierarchical structure, which has also been pursued by several authors^18,19,25^. Figure 1b,c shows top view images of the anodic layers grown on the aluminum surfaces previously etched during 22 min (CE-AL5 and CE-AL20). As it can be observed, the micro-step like structure morphology obtained with the chemical etching process was maintained after anodization with two different thicknesses. Anodic layer replicated the microtexturing and the growth of aluminum oxide was conformal. Furthermore, the roughness (Ra and Rt, as presented in Table 1) of the 5 and 20 μm thick anodic layers were similar to the roughness of the surface etched at 22 min. Therefore, a well distributed micro-step like structure similar to the original CE was maintained after the oxide layer growth up to 20 µm.

After attaining micro and micro-nano structures, two main strategies were followed to modify the composition of the surfaces. Both were based on the introduction of polyfluoroalkyl moieties. The first one was based on surface functionalization through FAS17 grafting. FAS17 grafting is a surface modification in which Al–OH terminal groups are replaced by Al–O–Si–(CH_2_)_2_–(CF_2_)_7_–CF_3_, known as self-assembled monolayer. Consequently, no major modification of the surface was observed, neither from the morphological point of view (see Figs. 1, 2 and 4) nor from the Ra nor Rt measured on the surfaces (see Table 1). The second one consisted in the application of a thin hybrid coating synthesized by sol–gel, with the presence of FAS17 in its formulation. This ⁓ 1.6 μm thick coating was composed of methacrylate, silica and zirconia with polyfluoroalkyl moieties covalently linked to the matrix through Si–C bonds. It has been demonstrated in previous works of authors^28,29^, that the application of sol–gel coatings on aluminum surfaces by dip-coating does not follow a conformational growth, since it contributes to the surface levelling as schematically represented in Fig. 2. As observed in Fig. 1d–i and Fig. 4, the relatively low thickness of the FAS17-hybrid coating allowed to maintain the micro-step like texture. However, the roughness of the FAS17-hybrid coatings on CE, CE-AL5 and CE-AL20 surfaces, compiled in Table 1, where Ra and Rt are shown, was slightly reduced in all cases due to its levelling effect.

### Analysis of the wettability

It is well known that the wetting of a surface by a liquid is affected by the roughness and morphology of the surface^30–32^. Indeed, an effective way to enhance the hydrophobic properties of a surface is to increase its surface roughness. In fact, in the case of a material with one of the lower known surface free energy, polytetrafluoroethylene (PTFE), superhydrophobic properties have only been achieved when combined with high roughness^33^. In this sense, the micro-step like structure processing on the aluminum surface, is a promising approach to produce amphiphobic surfaces^10^.

Surface functionalization through fluorine containing groups is an extended strategy for superhydrophobicity. Surfaces containing -CF_3_, -CF_2_- and -CF_2_-CH_2_- are among the materials with lower interfacial energy^34^ and the length of polyfluoroalkyl groups introduced in a coating has a direct effect on its hydrophobicity^35^. In this study, FAS17 molecules integration has been considered in two ways, one grafting the molecules on the aluminum surface and the other one, through a hybrid coating containing FAS17 molecules, representing about ⁓7.4% of the dried coating material.

**Figure 5:**
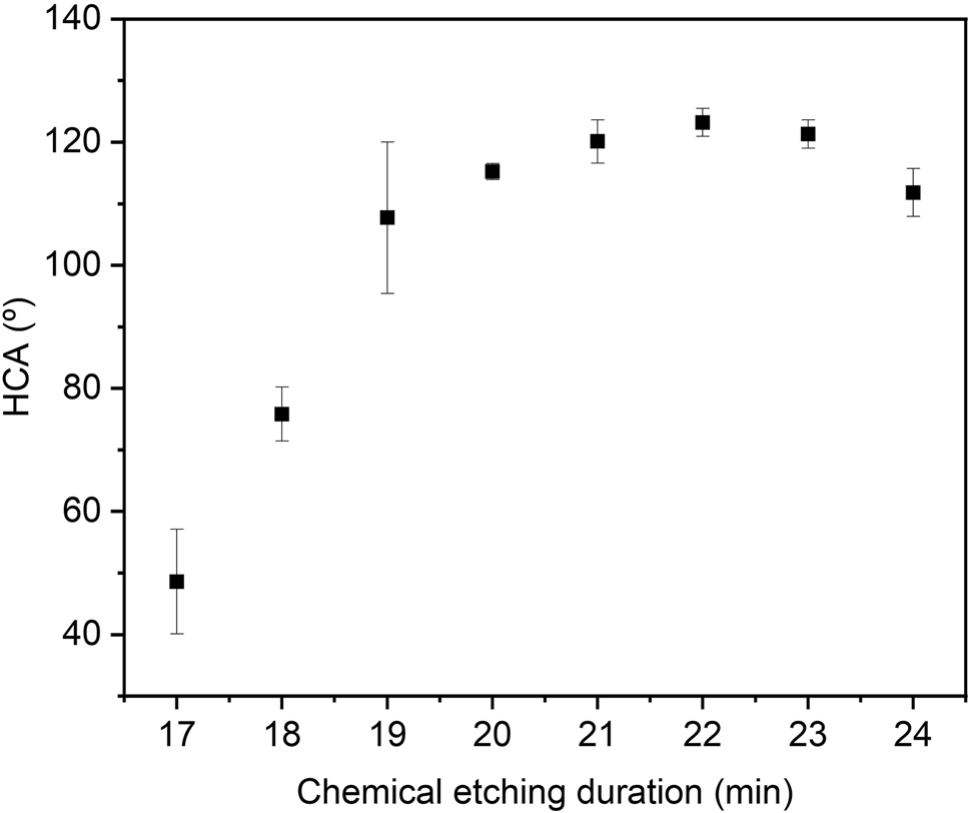
HCA measurement on chemically etched AA1050 with different duration after FAS17-grafting.

Wettability properties of the aluminum surface with micro-step like structures combined with FAS17-grafting have been studied with the aim to establish the influence of the etching duration on processed surfaces. As FAS17-grafting is a self-assembled monolayer of long-chained polyfluoroalkyl that does not have effect on the surface morphology, the WCA and HCA of FAS17-grafted chemical etched surfaces varied depending on chemical etching duration, as presented in Fig. 5. The surfaces were in all cases superhydrophobic although WCA was not quantitatively measured due instability of the droplet when contact angle was higher than 140° (droplet not holding on to the surface).

HCA of the FAS17-grafted and chemical etched surfaces at different duration was quantitatively analyzed. As observed in Fig. 5, an increase in HCA took place up to 22 min of etching duration, being the major change when increasing from 17 min, with HCA of 48° to 19 min, with HCA of 107º. However, the values up to 21 min showed relatively high dispersion, which can be in agreement with surface roughness measurements. Above 21 min etching, a slight HCA increase was observed, reaching the maximum HCA of 123° at 22 min etching with low HCA dispersion, in agreement with the homogeneous surface morphology observed.

The FAS17-grafted anodized surfaces showed again superhydrophobicity although WCA could not be quantitatively determined, resulting in a demonstration of the SHP behavior. The HCA for these surfaces was also extremely high (oleophobic), since it reached almost 120° for the FAS17-grafted CE-AL5 and CE-AL20, showing that anodizing up to 20 µm thick had a limited impact on sample wettability performance.

All the FAS17-grafted surfaces presumably presented a monolayer of polyfluoroalkyl moiety thanks to the reaction of the -OH groups of the surface with the alkoxy groups of the FAS17 resulting in a surface highly functionalized with covalent stable bonding of the polyfluoroalkyl group to the metal: Al–O–Si–CH_2_–CH_2_–(CF_2_)_7_–CF_3_.

Table 2 compiles the WCA and HCA values of the FAS17-grafted and FAS17-hybrid coated AA1050 after 22 min of chemical etching (CE), as well as after anodization processes (CE-AL5 and CE-AL20).

**Table 2:** Contact angle measurements with water (WCA) and hexadecane (HCA) of chemical etched and 20 μm-thick anodized AA1050 after FAS17-grafted and FAS17-hybrid coating treatments before and after NSST.

	Initial values				After 2016 h of exposure to NSST			
	FAS17-grafted		FAS17-hybrid coated		FAS17-grafted		FAS17-hybrid coated	
	WCA (°)	HCA (°)	WCA (°)	HCA (°)	WCA (°)	HCA (°)	WCA (°)	HCA (°)
CE	> 140	123.2 ± 2.3	137.7 ± 1.9	60.1 ± 1.5	88.7 ± 14	49.8 ± 10	53.8 ± 15.1	< 10
CE-AL5	> 140	119.2 ± 1.0	129.2 ± 4.2	55.4 ± 1.3	123.9 ± 4.6	115.7 ± 3.3	63.4 ± 13.9	< 10
CE-AL20	> 140	118.5 ± 1.9	130.8 ± 2.3	49.1 ± 5.8	> 140	123.3 ± 1.8	77.8 ± 4.3	< 10

In the case of the FAS17-hybrid coated surfaces, the material deposited was composed by a matrix of methacrylate-silica-zirconia, with low presence of polyfluoroalkyl moieties. Even with this low percentage of polyfluoroalkyl groups, although the achieved WCA did not reach superhydrophobicity, it was high, being close to 140º on CE and ⁓130º on CE-AL5 and CE-AL20, as presented in Table 2. However, the HCA was notably lower in comparison to FAS17-grafted, which could be attributed to the lower concentration of polyfluoroalkyl groups in the external surface, and the reduction of the roughness due to the levelling effect of the hybrid coating respectively.

### Mechanical properties

Microhardness measurements were carried out to evaluate the effect of the chemical etching and anodizing process on the mechanical properties of the bulk microtextured AA1050 alloy.

On the one side, it is important to assess how immersion in a strong acid solution and consequently created porosity and roughness affect the mechanical properties on both the surface and bulk material of the AA1050. On the other side, anodizing process, apart from providing corrosion protection to aluminum, usually allows to improve the hardness, abrasion and wear resistance of the aluminum surface.

**Figure 6:**
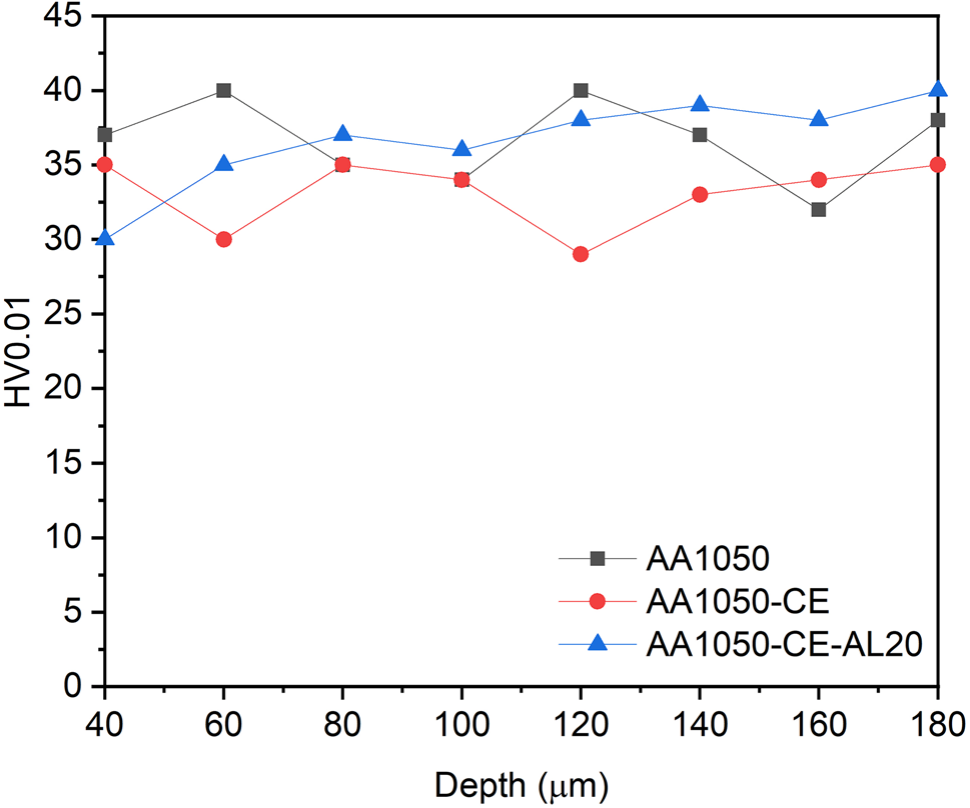
Cross section microhardness at several depths on pristine AA1050 and after chemical etching (CE) and 20 μm-thick anodic layer (AL20).

Firstly, the bulk material AA1050 before and after treatments was studied through conventional Vickers microhardness testing by optical evaluation of indentation tracks performed along cross-section. This value is designated as Vickers Hardness HV, as described in the ISO 6507 standard, and only takes into account the plastic deformation. Considering that the roughness of the microtextured samples showed a peak to valley distance between 40 and 50 µm, microhardness measurements starting at 40 µm depth were considered in this study. Figure 6 shows the results on the AA1050, CE and CE-AL20 samples at different distances from the surface. It can be observed that microhardness results are similar for the three materials, all falling between 30 and 40 HV0.01. Consequently, it was concluded that the bulk of the AA1050 was not affected by surface microtexturing.

**Figure 7:**
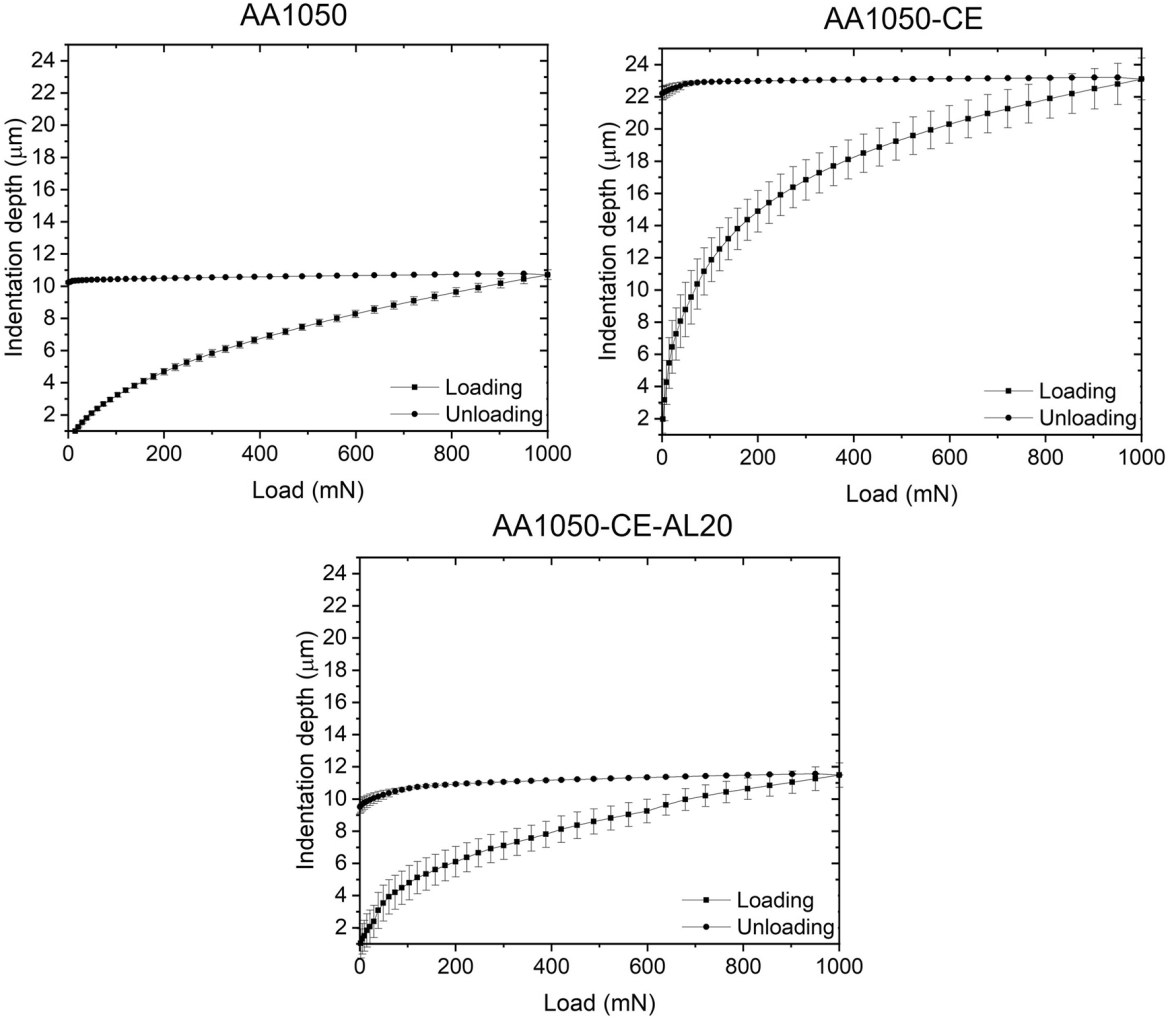
Dynamic Microhardness test results on pristine AA1050 and after chemical etching (CE) and 20 μm-thick anodic layer (CE-AL20).

Then, dynamic indentation testing according to ISO 14,577 standard was conducted on the surface of pristine AA1050, CE and CE-AL20 surfaces. This method takes into account both the elastic and plastic components of the deformation. Figure 7 shows the loading–unloading indentation curves corresponding to bare AA1050, CE and CE-AL20 and Table 3 shows the summary of dynamic microhardness results on tested samples. At maximum load, 1000 mN, indentation depth of pristine AA1050 was 10.7 ± 0.4 µm, while after chemical etching treatment, it grew up to 23.1 ± 1.6 µm. The indentation hardness measured at the maximum load, H_IT_, of AA1050 was of 330 ± 23 MPa and it was reduced up to 71 ± 10 MPa after chemical etching. Therefore, it was proved that chemical etching with HCl solution on AA1050 aluminum alloy had a negative impact on surface mechanical properties, which were considerably reduced. The indentation depth measured on the 20 µm-thick anodic layer was 11.4 ± 0.9 µm and the H_IT_ was 287 ± 47 MPa, in the same range of the pristine AA1050. Consequently, the detrimental effect produced on the surface mechanical properties after chemical etching, was counteracted by the deposition of the anodic layer.

**Table 3:** Maximum indentation depth (h_max_), indentation hardness (H_IT_) and modified elastic modulus (E*_IT_) of pristine AA1050, after chemical etching (CE) and 20 μm-thick anodic layer (CE-AL20).

	h_max_ (µm)	H_IT_ (MPa)	E*_IT_ (GPa)
AA1050	10.70 ± 0.37	330 ± 23	53 ± 2
CE	23.10 ± 1.59	71 ± 10	32 ± 1
CE-AL20	11.44 ± 0.93	287 ± 47	24 ± 1

Therefore, it can be concluded that after chemical etching, the affected area below the surface is restricted to the roughened area (approximately 50 µm depth) with no influence on the bulk of the material, according to the results obtained in the cross-section study.

### Corrosion resistance

Up to now, different works have studied the corrosion resistance of SHP aluminum surfaces through electrochemical testing^22–24^, although little information on their wettability properties after long exposure to corrosive environments has been found^21,25,36^. Herein, in order to evaluate the durability of processed superhydrophobic and oleophobic surfaces, their corrosion resistance was assessed under NSST, as well as the change of wettability after long exposure of 2016 h.

**Figure 8:**
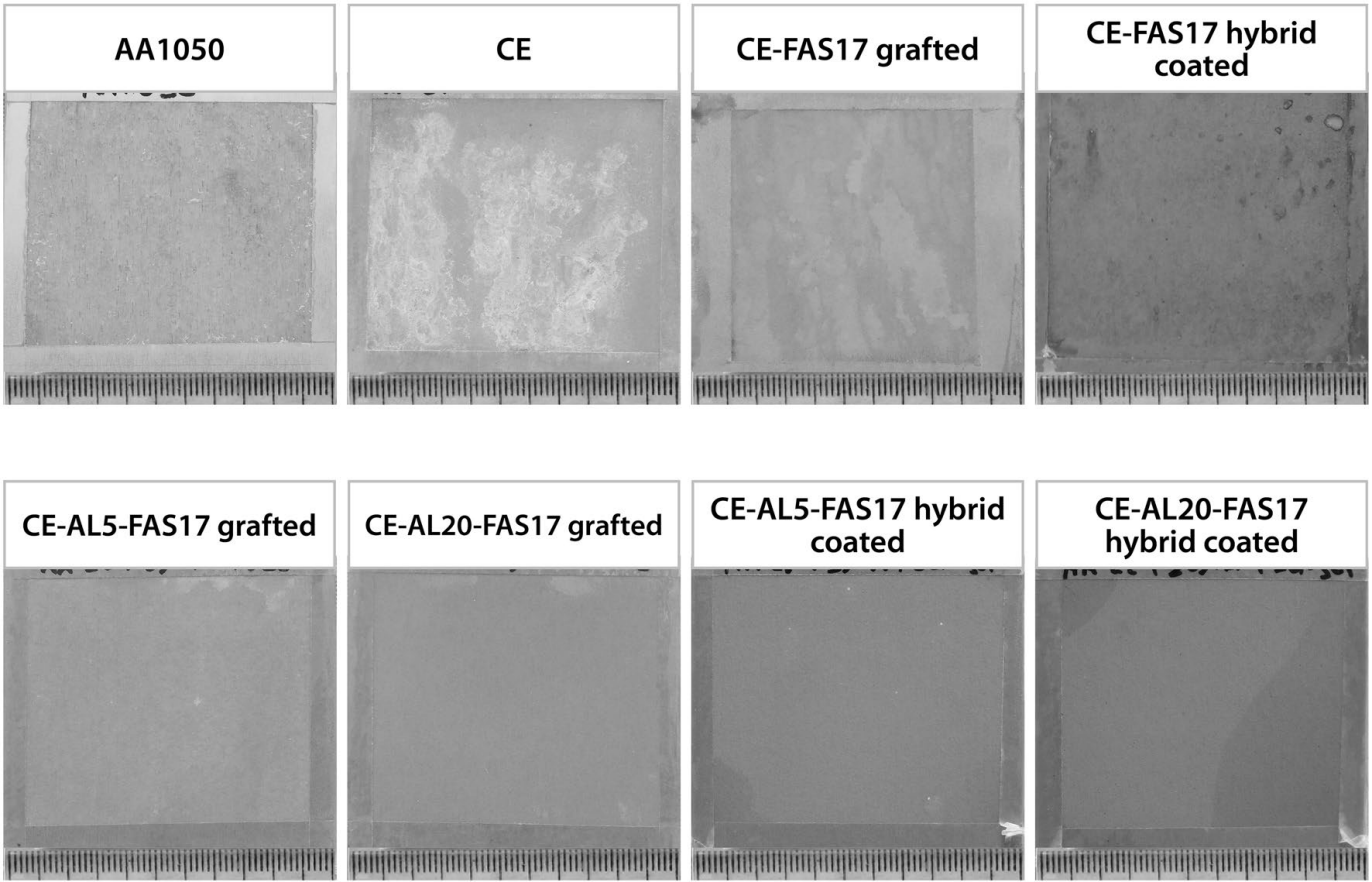
Images of pristine AA1050, chemical etched samples CE; chemical etched and fucntionalized samples CE-FAS17-grafted, CE-FAS17 hybrid coated; chemical etched, anodized and fucntionalized samples CE-AL5-FAS17 grafted, CE-AL20-FAS17 grafted, CE-AL5-FAS17 hybrid coated, CE-AL20-FAS17 hybrid coated after 2016 h of exposure to NSST.

The Fig. 8 shows the appearance of pristine AA1050, chemical etched AA1050 (CE), FAS-17 grafted CE and FAS17-hybrid coated CE, as well as FAS17-grafted and FAS17-hybrid coated anodized samples after 2016 h of exposure. The pristine and chemical etched AA1050 presented signs of corrosion after 24 h of exposure, in particular, white and grey corrosion products emerged on the aluminum surface. At the end of the test, the chemical etched AA1050 presented highly extended white corrosion products all over the surface. The chemical etching treatment alone, had a negative effect on the durability of AA1050.

Hybrid coatings prepared by sol–gel method have been widely used for corrosion protection of metals. In particular, formulations based on mixtures of silicon and zirconium alkoxides in combination with organo-alkoxides such as MAPTMS have demonstrated corrosion protection of aluminum alloys highly susceptible to corrosion, such as AA2024^28^. In this case, the hybrid coating has been modified with FAS17 to also improve superhydrophobicity. Both chemical modifications (grafting and coating) with FAS17 on CE samples delayed the emergence of signs of corrosion. The surface of FAS17-grafted CE exhibited color changes during the test while the FAS17-hybrid coated CE presented color change and presence of corrosion products on the surface after 336 h of exposure. It can be concluded that both FAS17-grafting and FAS17-hybrid coating slightly protected the AA1050 after chemical etching.

Anodization treatment provides corrosion protection and hardness to aluminum parts. Although the properties of the anodic layer depend on the aluminum alloy, the electrolyte, and the anodizing parameters, industrial processes usually provide corrosion protection to aluminum surface.

According to MIL-A-8625F, the sealed anodized aluminum alloys must be exposed during 336 h to NSST in accordance with ASTM-B117, except that the surface shall be inclined 6 ± 2° from the vertical. The CE samples covered with anodic layers of two thickness, 5 and 20 μm, and then modified with FAS17-grafting and FAS17-hybrid coating did not present any corrosion failure after 336 h. Therefore, the exposure to the corrosive environment was prolonged up to 2016 h in order to observe differences among the different treatments. It is also important to envisage that the corrosion resistance required for anodized aluminum with a subsequent organic primer applied on top of the conversion coating is 2016 h in NSST, according to specifications MIL-P-85582B and MIL-PRF-23377 J. At the end of the test, the anodized CE-AL5 and CE-AL20 AA1050 both with FAS17-grafting and hybrid coating appeared intact, without signs of white corrosion. Therefore, the anodic layers of 5 and 20 μm demonstrated to protect against corrosion the chemical etched AA1050 surface.

To study the durability of SHP and oleophobic properties, WCA and HCA were measured on the FAS17 modified surfaces after the 2016 h of exposure to NSST and presented in Table 2. Considering the FAS17-grafting treatment, the chemical etched surface presented a considerable reduction of WCA and HCA after NSST exposure, falling up to 88.7 ± 14 and 49.8 ± 10, respectively. FAS17-grafted CE-AL5 showed a slight reduction in the WCA and HCA values, which were 123.9 ± 4.6 and 115.7 ± 3.3, respectively. Finally, outstanding result was obtained for FAS17-grafted CE-AL20 surface, which maintained the SHP and oleophobic properties after corrosion test. In the case of FAS17-hybrid coated surfaces, they presented a considerable reduction of WCA and HCA after NSST exposure. None of them maintained the hydrophobicity, since WCA was < 90° in all cases and the aged surfaces presented high affinity to hexadecane, since HCA was < 10° in all cases, even if no damage was observed visually.

Some works addressing the corrosion resistance of SHP aluminum surfaces suggested^36^ that even being SHP, the coating permeability may be the reason for the penetration of water into the coating after certain exposure time. This may cause defects on FAS17 treatments and the modification of the chemically microtextured surface due to corrosion products forming under the coating. This could be the reason for the reduction in wettability of FAS17-grafted 5 µm thick anodic layer, while the 20 µm thick anodic layer seemed to help maintaining the integrity of the chemically microtextured surface and the FAS17-graftig layer.

With the aim to assess the integrity of the FAS17 molecules on the surface, the variation of fluorine content on the FAS17-grafted CE-AL20 was studied by EDX analysis before and after NSST exposure together with the bare CE-AL20 for reference. As presented in Table 4, the results of surface composition in atomic percentage confirmed that fluorine concentration on the surface was not modified after the 2016 h exposure in NSST.

**Table 4:** EDX analysis of the chemical etched and 20 μm-thick anodized AA1050 (AL20) before and after FAS17-grafting, and FAS17-grafted sample after 2016 h of exposure to NSST.

	O (at.%)	F (at.%)	Al (at.%)	S (at.%)	Ni (at.%)
CE-AL20	51.17	–	37.91	3.67	7.26
CE-AL20-FAS17-grafted	43.69	9.25	37.35	3.41	6.3
CE-AL20-FAS17-grafted after 2016 h of exposure to NSST	52.47	9.71	31.87	2.73	3.22

Therefore, the combination of the FAS17-grafting and the 20 µm-thick anodic layer on the chemically microtextured aluminum surface protected the surface against corrosion and consequently favored the durability of the wettability properties after 2016 h of exposure to NSST.

## Conclusions

The design of a treatment for AA1050 surfaces with superhydrophobic and oleophobic durable properties has been successfully obtained in this work through the combination of chemical and industrial electrochemical treatments on the surface with chemically bonded polyfluoroalkyl moieties.

In particular, an optimized chemical etching with HCl allowed to attain a micro-step like structure maintained after the sulfuric anodization treatments (5 and 20 µm thick layer). Although the chemical etching treatment itself provoked the reduction of the surface mechanical properties of AA1050 due to the introduction of porosity and roughness, the anodic layer improved the surface hardness to values similar to pristine AA1050.

Concerning the chemical modification with FAS17 molecules, the growth of a self-assembled monolayer of the long-chained polyfluoroalkyl groups did not change the surface morphology, resulting in superhydrophobic and oleophobic surfaces. On the other side, the deposition of a thin layer of hybrid coating with lower presence of polyfluoroalkyl long groups in its structure, had a levelling effect reducing the surface roughness, and showed hydrophobicity although not in the same grade as FAS17-grafted surfaces. Such surfaces were not oleophobic.

The presence of anodic layer after chemical microtexturing in combination with FAS17 modifications, provided excellent corrosion protection after 2016 h of exposure to NSST. The FAS17-grafting on 20 μm-thick anodic layer deposited on chemically microtextured AA1050 maintained the superhydrophobicity and oleophobicity properties after 2016 h of exposure to NSST.

In conclusion, a scalable, simple and cost-effective chemical microtexturing combined with an anodic layer and FAS17-grafting allowed to attain superhydrophobic and oleophobic performance of AA1050, with mechanical properties similar to pristine AA1050 and outstanding durability of wetting properties under salt spray environments, favoring the qualitative jump required for such solutions to be introduced into industrial applications.

## Material and methods

### Materials

The substrate material used was 1-mm thick sheet of commercial cold-rolled wrought AA1050 provided by Alustock S.A.

HCl (37 wt.%) was purchased from Scharlab and used to prepare 3 M solution in deionized water.

Granular mixture of sodium bifluoride and ferric sulfate from Turco Española, S.A. as well as HNO_3_ (67%) from Sigma-Aldrich (St. Louis, MO, USA) were used to prepare deoxidation bath.

Absolute ethanol (99.9% purity) was purchased from Scharlab S.L. 1H,1H,2H,2H-perfluorodecyltriethoxysilane (FAS17, 97% purity) was purchased from Sigma-Aldrich. Tetraethyl orthosilicate (TEOS, purity 98%) was purchased from Acros Organics. Methacryloxy propyl trimethoxy silane (MAPTMS, ≤ 100%) was purchased from Evonik Industries AG. Ethylene glycol dimethacrylate (EGDMA, purity 98%, with 90–110 ppm mono-methyl ether hydroquinone as inhibitor) and 2,2′-azobis(2-methyl- propionitrile) (AIBN, purity 98%) as thermal initiator were purchased from Sigma-Aldrich and used as received. Zirconium (IV) n-propoxide (TPOZ, 70 wt.% solution in 1-propanol) was purchased from Acros Organics and methacrylic acid (MAAH, purity 99.5%) from Scharlab. All of them were used as received.

### Surface treatments

#### Microtextured surface processing

For the surface microtexturing process, the AA1050 plates were first cleaned in acetone and dried by nitrogen blow. Chemical etching was applied by dipping the aluminum plates into a 3.0 M HCl solution at room temperature for 17 to 24 min. Then, they were rinsed with deionized water and dried in a nitrogen blow. At the end, a deoxidation step was applied in an acid bath composed by Fe_2_(SO_4_)_3_ (60–108 g/L), NaHF_2_ (< 12 g/L) and HNO_3_ (7 g/L) in distilled water at room temperature during 5 min. Then they were rinsed in deionized water and dried with compressed air (the AA1050 specimens treated during 22 min are referred as CE).

Anodizing process of chemically etched AA1050 was carried out by Anesdur S.L. (Ordizia) in sulfuric acid medium according to internal procedures that allowed to obtain coatings which meet the requirements of the MIL-A-8625 TYPE II, CLASS 1 standard. Anodic layers with thicknesses of 5 and 20 μm were grown (these treatments are referred as AL5 and AL20 respectively).

#### Surface functionalization treatments

For the polyflouroalkyl (FAS17) grafting, firstly FAS17 was diluted in ethanol solution (1 wt.%). Then, the AA1050 specimens were dipped during 2 h and cured for 30 min at 100 ºC to obtain a self-assembled monolayer for surface functionalization (referred as FAS17-grafted).

For the deposition of a polyflouroalkyl (FAS17) modified hybrid sol–gel coating, firstly TEOS, MAPTMS and FAS17 were mixed in ethanol media and after homogenization, aqueous solution HNO_3_ 0.01 N was added. The resultant solution was stirred during 3 h at 80 °C. Once the mix reached room temperature, EGDMA and AIBN were added. Separately, TPOZ diluted in n-propanol was admixed with MAAH and stirred during 1 h. Then, both parts were slowly admixed together and aqueous 0.01 M HNO_3_ solution was added to obtain the final sol with a FAS17 content of 3.8 wt.%. Ultimately, the AA1050 specimens were immersed in the sol and withdrawn at controlled rate of 15 cm /min. After deposition, sol–gel coatings were treated at 150 °C during 2 h and a thin layer of ⁓1.6 µm thickness was obtained (referred as FAS17-hybrid coated).

### Characterization

The morphology of microtextured and treated surfaces was examined with images obtained by scanning electron microscopy (SEM) using a low-vacuum JEOL JSM 5910 LV equipment with an added microprobe INCA Act-X for Energy Dispersive X-Ray Spectroscopy (EDX) analysis of the surface. Secondary electron and backscattered electron images were collected at 20 kV.

Roughness measurements were performed using a contact profilometer Dektak 150, according to ISO 4288. Six 5-mm length scans were taken on each sample using a 2-µm radius stylus, 1 mg force and lengthwise resolution of 0.111 µm. Arithmetic average roughness, Ra, was calculated from the central 4 mm of the scan using short and long pass filter cut-off of 800 µm. The maximum height of the profile, Rt, corresponded to the difference between the highest and the lowest peaks of the profile roughness above or below the mean line.

The static contact angle of water (interfacial energy 72.8 mN/m) and hexadecane (interfacial energy 27.5 mN/m) was measured using a goniometer (Digidrop MCAT and OCA 15 EC Dataphysics) by depositing 20 μL-droplets on each microtextured and functionalized specimen. Droplets were placed on three different spots on each sample and the image analysis was performed to determine the contact angles.

 Microhardness was measured on surface and the bulk of different samples using different techniques.

On bulk material, conventional Vickers microhardness was measured at different depths from the aluminum surface. For this purpose, metallographic samples were prepared embedding and polishing the cross-cut surface of the different specimens. The test conducted was based on the ISO 6507 standard and consisted in performing an indentation of a square pyramidal standard Vickers indenter with a load of 10 g (HV0.01) and the ulterior analysis of the area of indentation track using optical microscope. Microhardness was measured in the area below the microtextured surface. Two measurement chains were carried out from the surface to the inner area to keep 40 μm distance among indentation marks.

On surface, dynamic microindentation testing based on the ISO 14,577 standard was conducted using a Fischerscope H100 equipment with a square pyramidal standard Vickers indenter operating at linear loading rate. Load and unload cycles were registered by scanning 40 points from 0.4 to 1000 mN. A minimum of 10 measurements were performance on each sample. Indentation hardness, H_IT_, was calculated according to the ISO 14,577 standard, based on the Oliver-Parr method. Modified elastic modulus, E*, was calculated using the linear part of the unloading curve and correcting the elastic deformation of the indentation according to Eq. 1, where F_max_ is the maximum indentation load; h_max_ is the maximum indentation depth; h_r_ is the point of intersection of the tangent of the unloading curve at F_max_ with the x-axis in the load vs. indentation depth plot; and A is the contact area.

1 $${E}^{*}=\frac{{F}_{max}}{2({h}_{max}-{h}_{r})}\sqrt{\frac{\pi }{A}}$$

Corrosion performance was evaluated by exposure of specimens in neutral salt spray fog test (NSST). The test was carried out in a salt spray chamber C&W Model SF/1000/CCT. The test conditions were in accordance with ASTM B117, which maintained a salt spray fog environment coming from a 5% NaCl solution at 35 ºC. Panels were exposed supported at 6º from the vertical in accordance with aeronautic specifications (MIL-A-8625F, MIL-P-85582B and MIL-PRF-23377 J) up to 2016 h. The size of the tested panels was of 50 × 50 × 1 mm, and the edges of all the specimens were masked using a tape in order to cover the possible defective zones of the coatings at the edges. The test specimens were periodically checked during testing and photographs were taken for their analysis.

## Data Availability

The datasets used and/or analyzed during the current study are available from the corresponding author on reasonable request.
